# Exploring the relationship between mean performance and within-person variability on smartphone-based cognitive testing in adults across the lifespan

**DOI:** 10.1038/s44277-025-00036-x

**Published:** 2025-06-19

**Authors:** Laura M. Campbell, Andrea M. Weinstein, Ashley Henneghan, Emily W. Paolillo, Robert Ackerman, Jessica Bomyea, Colin A. Depp, Philip D. Harvey, Amy E. Pinkham, Elizabeth W. Twamley, Raeanne C. Moore

**Affiliations:** 1 Department of Psychiatry, UC San Diego, San Diego, CA USA; 2 Department of Psychiatry, and The Clinical and Translational Science Institute, University of Pittsburgh, Pittsburgh, PA USA; 3 School of Nursing, University of Texas at Austin, Austin, TX USA; 4 Department of Neurology, Memory and Aging Center, University of California, San Francisco, Weill Institute for Neurosciences, San Francisco, CA USA; 5 Department of Psychology, School of Behavioral and Brain Sciences, The University of Texas at Dallas, Richardson, TX USA; 6 Center of Excellence for Stress and Mental Health and Research Service, VA San Diego Healthcare System, San Diego, CA USA; 7 Psychology, VA San Diego Healthcare System, San Diego, CA USA; 8 Department of Psychiatry and Behavioral Sciences, University of Miami Miller School of Medicine, Miami, FL USA; 9 Research Service, Miami VA Healthcare System, Miami, FL USA

**Keywords:** Risk factors, Geriatrics, Diagnosis

## Abstract

Cognitive performance is classically measured through measures of central tendency. However, intraindividual cognitive variability (IIV) also holds important information about cognitive functioning. Smartphone-based ecological momentary cognitive testing (EMCT) can capture IIV across days. This study examined predictors of IIV, including demographics, affect, and mean performance, in EMCTs completed on the NeuroUX platform among two US-based samples: adults and older adults with high rates of mild cognitive impairment. The adult sample (*n* = 375) completed eight EMCTs assessing memory, processing speed, executive functioning, and working memory; each test was self-administered five times over ten days. The older adult sample (*n* = 94) completed three EMCTs assessing memory, processing speed/executive function, and working memory at three difficulty levels; each test at each difficulty level was self-administered five times over thirty days. Mean performance demonstrated the strongest association with IIV across groups. In the adult group, better mean performance was associated with less variability on tests of memory, executive functioning, and two out of three tests of processing speed. On tests of working memory, better mean performance was associated with greater variability, possibly due to the difficulty of these measures at higher performance levels. In the older adult sample, better mean performance was associated with two of three versions of the memory and working memory EMCTs and all difficulty levels of the processing speed/executive function task. Better average performance was associated with greater consistency across most measures. Broader and more diverse data collection methods like EMCT can provide valuable insights into cognitive functioning beyond traditional mean-based measures.

## Lay summary

This study used smartphone-based mobile cognitive tests to explore how consistently people perform across days, which is called intraindividual variability. Participants included two groups: adults and older adults, many with mild cognitive impairment. Results showed that individuals who performed better on average, in both groups, tended to have more consistent scores across days. These findings suggest that mobile cognitive testing may be a tool to provide insight into cognitive functioning, such as cognitive variability.

## Introduction

Traditional paper and pencil neuropsychological testing has long been the gold standard for cognitive assessment. Neuropsychological tests were developed to detect cognitive impairment following illness or injury, rather than subtle changes in cognition such as in the preclinical phases of a disease. Additionally, standard neuropsychological testing occurs in a controlled environment with administration by trained technicians, which does not fully mimic the cognitive demands of everyday life. Thus, there is need for neuropsychologists to develop and validate cognitive assessments that better reflect real-world cognitive abilities and are sensitive to more subtle, within-person changes [1].

One way to assess everyday cognitive functioning is with mobile sampling methods adapted from ecological momentary assessment (EMA). Ecological momentary cognitive testing (EMCT) borrows from EMA methods to include brief objective cognitive assessments that occur in daily life [2]. EMCT allows multiple “snapshots” of cognitive performance that can be measured over the course of days to understand the dynamics of everyday cognition and how cognition fluctuates with other life factors (e.g., stress, activities) across time [3–5].

One of the strengths of EMCT is that both intraindividual mean and variability in cognitive performance can be indexed by multi-day, burst assessment data. Intraindividual variability (IIV) is defined as within-person fluctuations in behavioral performance. From a cognitive perspective, IIV can be measured either as variability across multiple test scores within the same evaluation (dispersion), or as variability in performance of the same task across multiple time points (inconsistency). Both fluctuations in accuracy and reaction time can be used for measuring IIV. Data from traditional neuropsychological evaluations show that IIV across different neuropsychological tests within a single testing session is increased in psychiatric (e.g., severe mental illness [6, 7]), neurological (e.g., traumatic brain injury [8], multiple sclerosis [9], neurodegenerative diseases [10–12]), and medical conditions (e.g., HIV [13]; breast cancer survivors [14]). For example, in the context of Alzheimer’s disease (AD), greater within session but across test IIV predicts future decline and may be a more sensitive marker of decline than mean performance [11]. IIV is also sensitive to the mild changes that occur in cognitive aging, a phenomenon that has been established for many years [15, 16]. There are several ways to measure IIV such as between test variation as described above, and inter-trial reaction time variability on measures of processing speed. IIV measured by inter-trial reaction time variability shows a U-shaped association with age such that variability is highest in childhood, reaches a nadir in early adulthood, and increases again in mid to late life [15, 17, 18]. Additionally, IIV is elevated in persons with high risk of AD [10, 11], suggesting that IIV may be an important and distinct marker of cognition.

Traditional neuropsychological measures of IIV have weaknesses. Testing sessions typically last hours and are not always accessible, which makes measuring IIV across tests (dispersion) time intensive. Moreover, traditional neuropsychological testing often compares variability across *different* measures within a testing session that often lack a shared normative sample, potentially reducing precison. For example, many of the most most recent and popular tests in neuropsychological measures [19] (e.g., Wechsler Adult Intelligence Scale-IV/5, California Verbal Learning Test II/III, Halstead Reitan Battery) are not cross-normed with one another. Lastly, IIV from traditional neuropsychological measures derived across tests given within a few hours on the same day lacks the potential for examining day-to-day variations. EMCT-derived IIV can be calculated across multiple days and times, making it potentially more sensitive to conditions with known temporal variations, such as circadian disruption in AD [20, 21] and diurnal variation in depression [22, 23]. Despite its appeal, research on EMCT-derived IIV is limited, with only a handful of published studies to date, primarily in the context of cognitive aging and AD [24–26]. Preliminary research demonstrates that EMCT-derived IIV has potential as a marker of cognitive vulnerability as, for example, it can distinguish mild cognitive impairment (MCI) from normal cognitive functioning [25].

Building on this emerging evidence and addressing current gaps in the literature, the present study aimed to further characterize the correlates of EMCT-derived IIV and evaluate it across diverse adult populations. Therefore, this study examined how demographic factors, affect, and mean performance correlates with EMCT-derived IIV collected via the NeuroUX platform over several days. These correlates were first examined in a large adult sample aged 20–79 years, participating remotely. To examine if these correlates extend beyond a general adult sample, we replicated these analyses in a multi-site study of older adults with high MCI rates, aiming to examine a broader range of cognitive performance. A secondary goal in this replication sample was to assess the extent to which EMCT IIV was associated with traditional neuropsychological testing performance.

## Patients and methods

The two samples included in this study were drawn from independent research protocols with distinct aims and study designs. We leveraged available data from each to examine the replication and generalizability of findings across samples. While there is some protocol overlap, protocol differences reflect study-specific decisions tailored to the target populations and research questions. Data preprocessing procedures (e.g., exclusion criteria for EMCT data, as detailed below) were as similiar as possible to support comparability.

### Adult sample participants and procedures

The methods of this study have been previously published in Paolillo et al. [27]. Three hundred and ninety-four participants were recruited through Prolific, an online research platform. Participants provided informed consent for this study through this platform. Verified and eligible participants enrolled in the study. Inclusion criteria included being between the ages of 20 and 79 years old, being born and residing in the United States, and speaking English as their first language. There were no other specific exclusion criteria. Participants’ data were fully anonymized during the data collection process; researchers cannot access identifiable information through the Prolific platform. This study received exempt status approval from the UC San Diego Institutional Review Board (IRB). No participants indicated that they had received a diagnosis of MCI or dementia. Demographic characteristics from this sample can be found in Table 1.

**Table 1:** Participant characteristics.

	Adult Sample	Older Adult Sample
	(*n* = 375)	(*n* = 94)
Demographics		
Age	45.1 (16.1)	71.0 (7.0)
Sex (male)	182 (48.5%)	32 (34.0%)
Race		
African American/Black	46 (12.3%)	7 (7.4%)
Asian	23 (6.1%)	—
“Other”	19 (5.0%)	—
Mixed Race	28 (7.5%)	2 (2.1%)
White	259 (69.0%)	85 (90.4%)
Ethnicity (Hispanic, Latino/a)	27 (7.2%)	13 (13.8%)
Years of Education	14.7 (2.4)	16.0 (2.3)
Smartphone Type		
iOS	176 (46.9%)	57 (60.6%)
Older Android (≤Android 12)	118 (31.4%)	37 (39.4%)
Newer Android (≥Android 13)	81 (21.6%)	—
Percentage of the EMCT sessions completed	87.6 (20.0)	88.6 (11.3)
Average depression/sadness rating (out of 7)	2.1 (1.3)	1.4 (0.8)

*EMCT* ecological momentary cognitive testing.

Over 10 days, participants completed eight NeuroUX EMCTs five times (i.e., four different EMCTs once per day) and a brief EMA survey on their personal smartphones. These procedures took between 8–10 min each day and could be completed between 7 am and 8 pm. Alternate forms were used for the EMCTs in each session [27].

### Adult sample EMCT and EMA measures

The eight EMCTs included in the general adult sample are described in detail in Paolillo et al. [27] and included Memory List (recognition memory), Memory Matrix (visual working memory), Matching Pair (speed of information processing), Quick Tap 1 (speed of information processing), Quick Tap 2 (response inhibition), Odd One Out (visual working memory), CopyKat (visual working memory), and Hand Swype (cognitive flexibility). Raw scores for Quick Tap 1 and 2, Odd One Out, and Hand Swype represent the median reaction time of the trial whereas all other raw scores generally represent total correct. Raw scores from the EMCT measures were transformed to T-score distributions (not demographically corrected) using the means and standard deviations published for this sample in Paolillo et al. [27], with greater T-scores indicating better performance for all measures. Transforming raw scores into a T-distribution allows for standardization across tasks and places scores on a common metric to facilitate comparison across EMCTs.

#### EMA

Participants responded to EMA questions related to affect, substance use, and contextual factors (e.g., location). To assess negative affect, participants rated their current level of sadness/depression from 1 = *not at all* to 7 = *extremely* at each session, with averages calculated to explore the association between mean negative affect and IIV. Participants also reported alcohol or cannabis use earlier in the day.

#### Exclusions of EMCT trials

After reviewing the data, one participant performed at the floor on all EMCT tasks and was excluded from analysis. Prior to calculating the mean or IIV, outliers on *specific trials* that were thought to be reflective of invalid performance were removed as in Paolillo et al. [27]. If participants reported any alcohol or cannabis use in the past two hours or four or more drinks at any time that day, then EMCT trials were considered invalid for that day. After calculating IIV, if IIV was greater >3 interquartile ranges above the sample’s median, these were considered IIV outliers and thought to reflect variability due to non-cognitive factors (e.g., variable effort). Eighteen participants did not have four valid administrations of any test and were therefore excluded. In total, 375 participants were included in this analysis.

### Older adult sample participants and procedures

To examine if correlates found in the general adult sample replicate and extend to a more clinically-based population, we also examined an older adult sample with and without cognitive impairment. The methods of this study focused on older adults who were either cognitively normal or had MCI (any subtype) have been published previously [28]. Data from 94 participants were collected from The University of Texas at Dallas, UC San Diego, and the University of Miami Miller School of Medicine. Inclusion criteria included being aged 50 and over, English-proficiency, and a familiar other who could serve as a study informant. Exclusion criteria included self-reported history of medical or neurological disorders that may affect brain function, dementia (i.e., clinical diagnosis or falling below the established cutoff scores on the MoCA [29]), self-reported head injury with loss of consciousness ≥15 min, vision or hearing impairment that would interfere with their ability to complete the study, intellectual disability (i.e., WRAT-4 Reading subtest standard score < 70), current substance use disorder, and history of a psychotic or bipolar disorder. Participant characteristics can be found in Table 1.

Procedures were approved by the IRBs of each respective university, and all participants provided written informed consent. Participants completed a baseline assessment to gather sociodemographic information and neuropsychological testing. As the study intended, about half of participants met criteria for any subtype of MCI using Jak/Bondi critera [30]. To mirror the general adult study, we chose to examine cognition longitudinally rather than by MCI status in order to examine if findings replicate in a broader range of cognitive abilities. Due to the evolving nature of COVID-19 restrictions, the baseline assessment was administered in person (32% of participants) and remotely (68% of participants). Additional details on COVID-19 adaptations can be found in Moore et al. [28].

Eligible participants then participated in EMCTs and an EMA survey via the NeuroUX platform for 30 consecutive days. Participants received training on the EMCT protocol and completed a practice EMA survey and mobile cognitive testing session. Participants had the option to use their own personal smartphone or could borrow a study-owned Android smartphone (*n* = 6). Over the next 30 days, participants were sent text message notifications to take EMA surveys three times per day. Every other day, the surveys were paired with EMCTs (i.e., three different EMCTs every other day).

### Older adult EMCT, EMA, and traditional neuropsychological measures

Details on these tests and the feasibility and validity of these tests in this sample are described in detail in Moore et al. [28]. The three different EMCTs in this study were Memory List (recognition memory; easy condition: 6-words; medium condition: 12-words and identical to the version in the normative sample; hard version: 18-words), Memory Matrix (visual working memory; easy version: 6 tiles; medium version: 12 tiles; hard version: 18 tiles), and Color Trick based on the classic Stroop paradigm (easy version: “Meaning-to-Meaning” to match the same words regardless of ink color testing speed of information processing; medium version: “Meaning-to-Color” to match the ink color to the word measuring inhibition; hard version: “Yes-No Mechanic” determining if the ink color matches a word measuring inhibition). For Memory List, scores were indicative of percent correct (i.e., 0–100%), and for Memory Matrix and Color Trick, scores ranged from 0–27, with greater scores indicating better performance. The EMCTs were counterbalanced by test type and difficulty level, resulting in a total of 5 easy, medium, and hard conditions of each of the three mobile cognitive tests. T-scores are not yet available for most of these EMCTs, so raw scores were utilized for these analyses.

#### EMA

EMA items to assess average negative affect and alcohol and cannabis use were the same as in the adult sample described above.

#### Exclusions of EMCT trials

Exclusions due to alcohol or cannabis use followed the same methodology as described for the adult sample. Following the same methodology of the adult sample, if an IIV calculation was >3 interquartile ranges above the median, then this IIV estimate was thought to reflect invalid performance and was removed from analysis. All 94 participants included in the study had at least one estimate of IIV.

### Traditional neuropsychological measures (lab or remote administered at baseline)

Participants in the older adult sample completed a neuropsychological exam at baseline. For the purposes of this analysis, we chose to examine a subset of tests measuring general cognition (Montreal Cognitive Assessment-BLIND version 7.1 (MoCA-BLIND); general cognition and screener for the presence of dementia), premorbid IQ (WRAT-4 Word-Reading subtest), and four additional measures most analogous to the EMCT measures as in the initial Moore 2022 validation paper [28]: Hopkins Verbal Learning Test – Revised (HVLT-R) immediate recall, delayed recall, and recognition (verbal learning and memory); Brief Visuospatial Memory Test – Revised (BVMT-R) immediate recall, delayed recall, and recognition (visual learning and memory); WAIS-IV Letter-Number Span Test (working memory); and all subtests from the D-KEFS-Color Word Interference Test (processing speed, inhibition, and inhibition/switching). Raw scores were used to examine the correlations between IIV and traditional neuropsychological tests except for the WRAT-4, in which we used the standard score, which adjusts for age.

### IIV

For both samples, IIV was calculated three ways: root mean square of successive differences (rMSSD) across five valid administrations, which accounts for gradual shifts in mean (e.g., due to practice effects); standard deviation (SD) from five valid administrations; and SD from the first four valid administrations. These were compared to determine which IIV estimate should be used, given that there is no “gold standard” approach to calculating IIV on EMCT. Coefficient of variation (CoV) was not considered. The CoV calculation includes the overall mean in the denominator. This study specifically examines associations with overall performance and variables (e.g., age, education) often associated with average performance. The CoV calculation has also been shown to amplify the IIV at low levels of mean [31].

### Statistical analyses

Multivariable linear regressions were conducted to examine associations between IIV (the outcome) and demographics including age, sex, race/ethnicity (dichotomized to non-Hispanic white and all others; restricted numbers of minoritized individuals did not allow for additional subgroup analyses), and years of education (continuous), as well as average negative affect rating reported on EMA, and mean performance on the EMCT. Type of phone was included as a covariate given known differences in smartphone performance regarding EMCTs [27, 32, 33]. Smartphone type was categorized into three groups: iOS models, “Older Android” (≤Android 12), and “Newer Android” (i.e., ≥Android 13). This grouping of different phones was based on differences published in Paolillo et al. [27]. These analyses were done twice: once with the adult sample and then replicated in the older adult sample. Assumptions of linear regressions were checked. For some IIV outcomes, residuals were skewed. Therefore, for these analyses, a log-transformed outcome was utilized. Log-transformed IIV was utilized for QuickTap 1, Odd One Out, Hand Swype, and Memory List for the adult sample and 6-Word Version of Memory List, 12-Tile version of Memory Matrix, and all Color Trick tests for the older adult group. Model statistics from these models therefore represent the statistics from log-transformed outcomes; however, for continuity across the figures we display non-log transformed values. Quadratic associations were examined for continuous predictors and retained in the models if significant at *p* < 0.05. For linear regression analyses, we corrected for multiple comparisons using the Benjamini Hochberg False Discovery Rate [34]. Lastly, in the older adult sample, we used Spearman correlations to examine if IIV was associated with traditional neuropsychological test performance.

## Results

### Associations between different methodologies to calculate intraindividual variability (IIV)

To inform our decision as to which IIV estimate to use for analyses, Spearman correlations were conducted to assess the association between IIV calculated from the SD from the first four valid EMCT administrations and IIV calculated from SD and rMSSD with all five EMCT administrations (i.e., since each trial was administered five times, this analysis required no missing data). There was a very strong correlation between SD calculated from the first four valid administrations compared to SD calculated from five assessments and rMSSD calculated from five assessments for most measures in the adult sample (see Table 2). More specifically, all were correlated at ρs > 0.76 except for Quick Tap 2 (ρ = 0.63, 0.68). Similarly, in the older adult group, all intraindividual SD calculated from four administrations were very strongly associated with SD and rMSSD calculated from five administrations (ρs > 0.81). These strong associations led to our decision to use the the four-administration statistic as our main outcome. This approach allowed for a larger sample size since some participants missed or had invalid data for all five administrations. Additionally, SD is more widely recognized and used in neuropsychology as a measure of IIV in traditional testing [13, 14], whereas rMSSD has been primarily utilized in psychophysiology for heart rate variability [35].

**Table 2:** Spearman associations between different intraindividual variability calculations.

	Association with SD calculated from 5 administrations	Association with rMSSD calculated from 5 administrations
Adult Sample with SD calculated from 4 administrations		
Memory List	0.84^**^	0.76^**^
Quick Tap 1	0.85^**^	0.88^**^
Quick Tap 2	0.63^**^	0.68^**^
Hand Swype	0.89^**^	0.92^**^
Odd One Out	0.77^**^	0.80^**^
CopyKat	0.91^**^	0.88^**^
Memory Matrix	0.82^**^	0.79^**^
Matching Pair	0.87^**^	0.84^**^
Older Adult Sample with SD calculated from 4 administrations		
Memory List 6-Word	0.91^**^	0.85^**^
Memory List 12-Word	0.92^**^	0.92^**^
Memory List 18-Word	0.81^**^	0.83^**^
Memory Matrix 6-Tile	0.91^**^	0.93^**^
Memory Matrix 12-Tile	0.89^**^	0.87^**^
Memory Matrix 18-Tile	0.87^**^	0.81^**^
Color Trick Meaning to Meaning	0.93^**^	0.87^**^
Color Trick Meaning to Color	0.97^**^	0.93^**^
Color Trick Yes-No Mechanic	0.97^**^	0.94^**^

*SD* standard deviation, *rMSSD* root mean square of successive differences.

**p < 0.01.

### Adult sample associations with IIV

Older age was associated with lower IIV for Memory List (β = −0.121, *p* = 0.030) and Memory Matrix (β = −0.183 *p* = 0.011), but only the Memory Matrix association remained significant after adjusting for multiple comparisons. Additionally, for Matching Pair, greater age was also significantly associated with lower IIV (β = −0.272, *p* < 0.001; significant after correcting for multiple comparisons), but there was also a significant quadratic effect of age (β = 0.119, *p* = 0.041) indicating the negative association weakened as age increased, but this was no longer significant after correcting for multiple comparisons. More education was significantly associated with greater IIV on Odd One Out median reaction time (β = −0.124, *p* = 0.013), and remained significant after adjusting for multiple comparisons. There was a significant quadratic relationship between higher education levels and increased IIV for CopyKat. Specifically, the association between education and IIV was not linear (β = 0.016, *p* = 0.741), but instead followed a quadratic pattern, where the effect of education on IIV increased as education levels rose (quadratic: β = 0.129, *p* = 0.013; remains significant with multiple comparison adjustment).

**Fig. 1:**
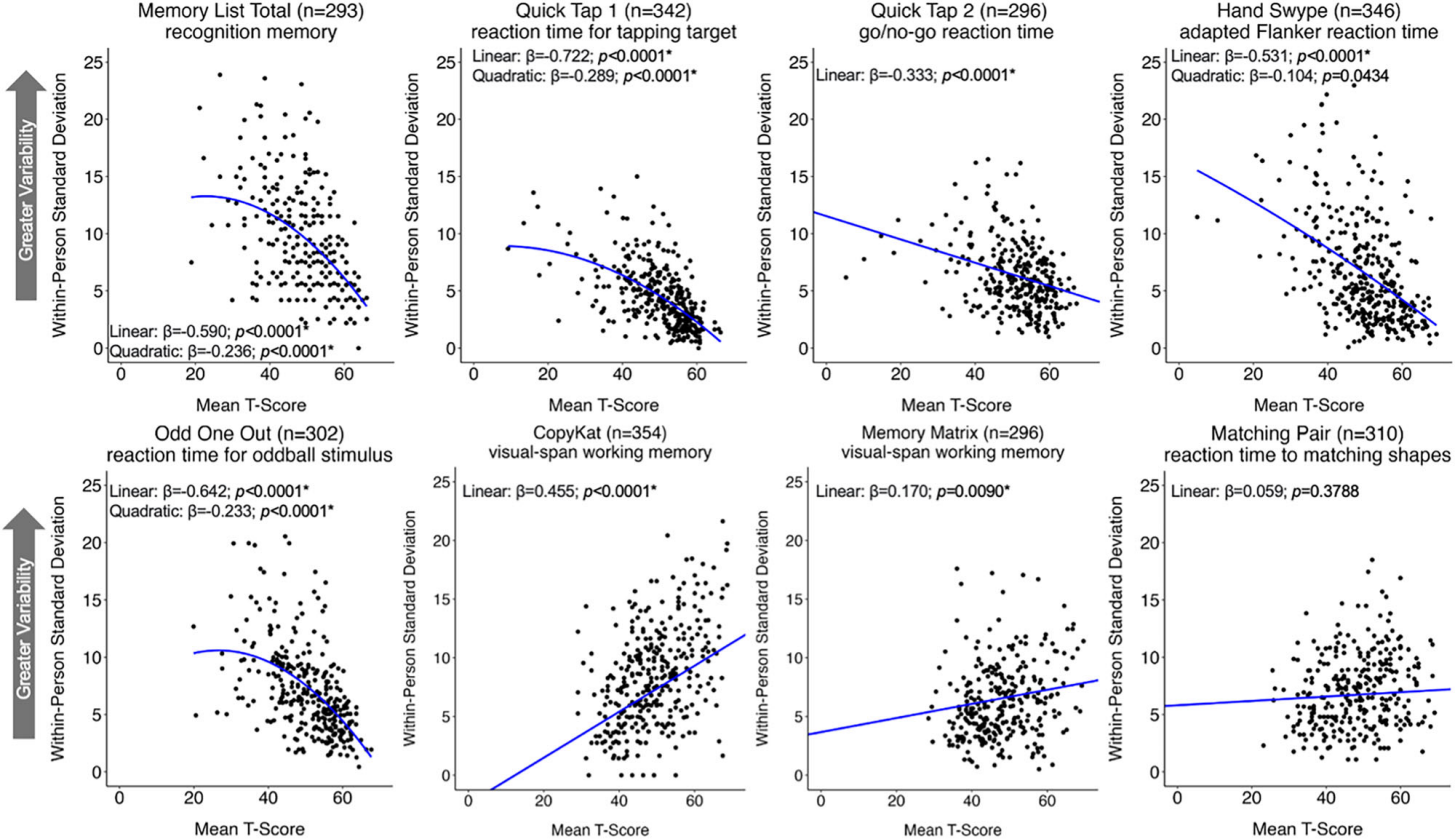
The associations between mean performance and individual variability on eight ecological momentary cognitive tests in the adult sample. Regression lines are adjusted for age (centered), sex (reference: female), race/ethnicity (reference: non-Hispanic white), education (centered), average negative affect (centered), and smartphone type (reference: iOS). T-scores are not demographically corrected. *significant after adjusting for multiple comparisons. Log-transformed IIV was utilized for Quick Tap 1, Odd One Out, Hand Swype, and Memory List model statistics.

Of all predictors examined, the greatest predictor of IIV for most tests was mean performance (i.e., demographically uncorrected average T-score). For Memory List, Quick Tap 1, Quick Tap 2, Hand Swype, and Odd One Out, worse mean performance was significantly associated with greater IIV. Additionally, Memory List, Quick Tap 1, Hand Swype, and Odd One Out also demonstrated a significant quadratic effect indicating that the slope (i.e., association) between IIV and mean performance is more steep (i.e., negative) at higher levels of mean performance. All remained significant after correcting for multiple comparisons except for the quadratic effect for Hand Swype. For CopyKat and Memory Matrix, better average performance was associated with greater IIV after correcting for multiple comparisons indicating a linear positive association. The only test in which IIV was not significantly associated with mean performance was Matching Pair. See Fig. 1 for the associations between mean performance and IIV and Supplementary Table 1 for comprehensive test statistics.

### Older adult sample associations with IIV

**Fig. 2:**
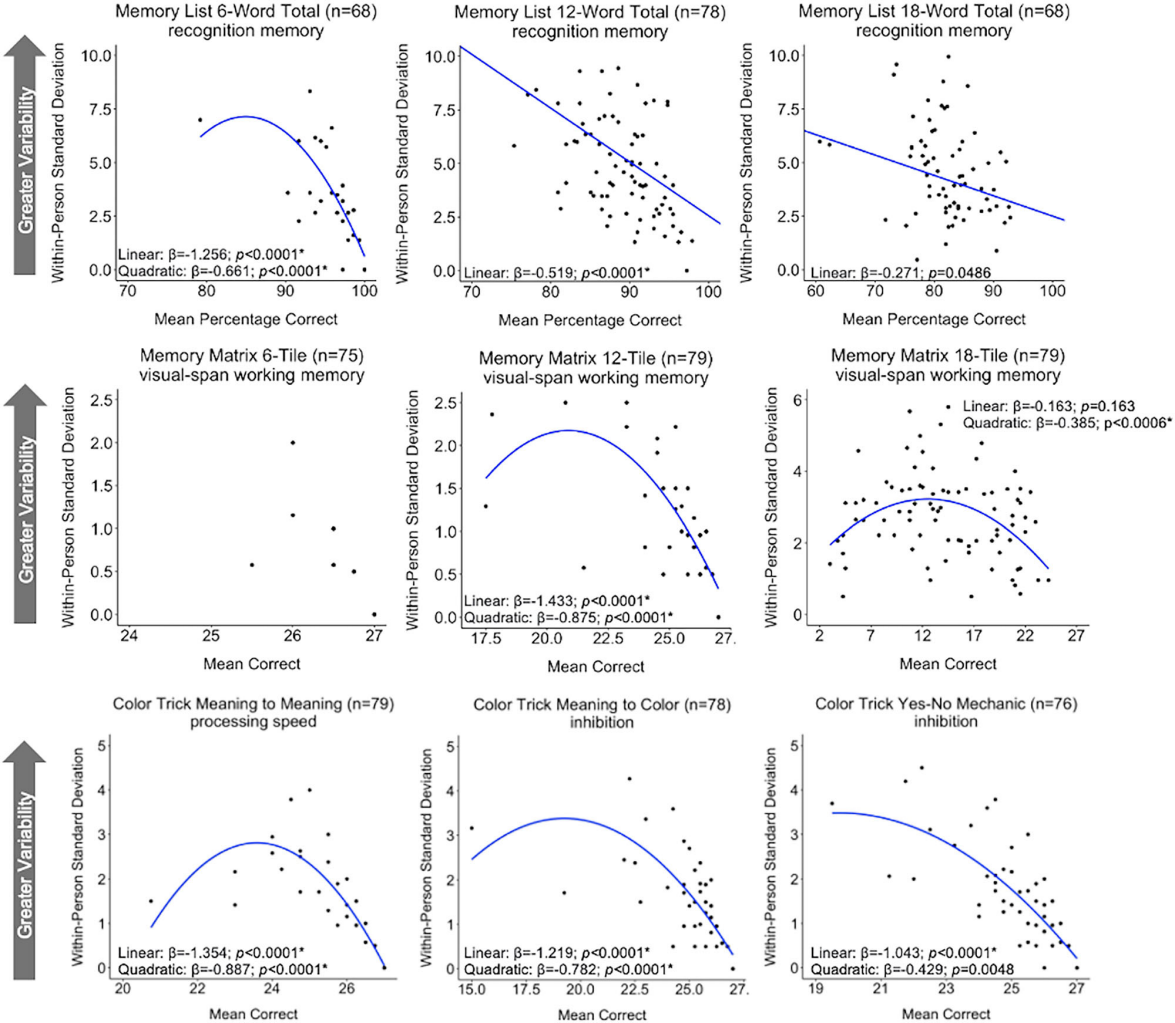
The associations between mean performance and individual variability on nine ecological momentary cognitive tests in the older adult sample. Regression lines are adjusted for age (centered), sex (reference: female), race/ethnicity (reference: non-Hispanic white), education (centered), average negative affect (centered), and smartphone type (reference: iOS). *significant after adjusting for multiple comparisons. Log-transformed IIV was utilized for 6-Word Version of Memory List, 12-Tile version of Memory Matrix, and all Color Trick model statistics.

Given the limited variability in scores for Memory Matrix 6-tile (i.e., most performed at the ceiling), there was very limited variability in IIV; thus Memory Matrix 6-tile was not examined in these analyses, but scores are presented in Fig. 2. Older age was associated with less IIV for Memory List 6-Word version (β = −0.222, *p* = 0.038) and Color Trick Meaning to Color (β = −0.227, *p* = 0.029), but they were no longer significant after correcting for multiple comparisons. Greater mean negative affect was associated with greater IIV for Memory List 12-Word version (β = 0.353, *p* = 0.001) and remained significant after correcting for multiple comparisons. Additionally, more education was associated with less variability on Memory Matrix 18-tiles version (β = −0.263, *p* = 0.024) but not after correcting for multiple comparisons.

The strongest association with IIV for the majority of tests was mean performance (i.e., average raw score). For the 6-Word version of the Memory List Test, the 12-Tile version of the Memory Matrix, and all Color Trick Tests, poorer average performance was significantly associated with greater variability. Additionally, these tests also demonstrated a significant quadratic association. The 18-Tile Memory Matrix test also demonstrated a significant quadratic effect, but the linear association was not significant indicating an inverted U-shape pattern where individuals with both low and high average performance showed lower variability, and those in the midrange demonstrated the greatest variability. All of these findings remained significant after multiple comparison correction. The 12-Word and 18-Word version of the Memory List Test and Quick Tap 2 demonstrated a significant linear association between worse average performance and greater IIV; the quadratic effects were not significant and removed from the model. However, after correcting for multiple comparisons, the 18-Word version of the Memory List Test was no longer significant. See Fig. 2 for the associations between mean performance and IIV and and Supplementary Table 2 for comprehensive test statistics.

### Older adult sample associations with traditional neuropsychological assessment

**Fig. 3:**
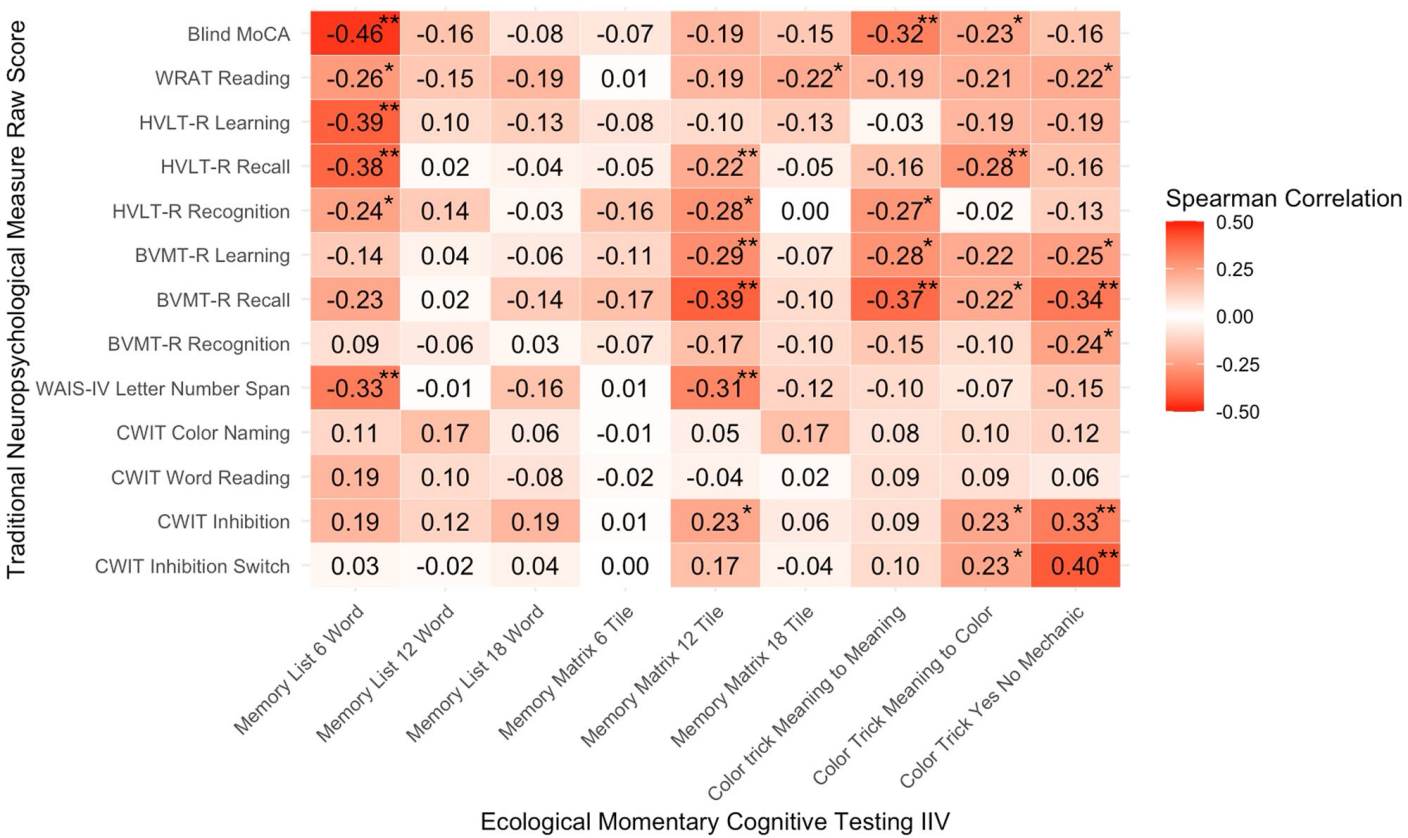
Spearman associations between intraindividual variability on ecological momentary cognitive testing and traditional neuropsychological measures in the older adult sample. MoCA Montreal Cognitive Assessment, WRAT Wide Range Achievement Test, HVLT-R Hopkins Verbal Learning Test-Revised, BVMT-R Brief Visuospatial Memory Test-Revised, WAIS-IV  Wechsler Adult Intelligence Scale-IV, CWIT Delis-Kaplan Executive Functioning System (D-KEFS) Color Word Interference Test. *p < 0.05; **p < 0.01.

Spearman correlations between each EMCT-derived IIV measure and the traditional neuropsychological assessment measures can be found in Fig. 3. Overall, there were more significant correlations with traditional neuropsychological measures for the 6-Word Memory List, 12-Tile Memory Matrix, and all measures of Color Trick. All significant correlations indicated a medium association and indicated that greater variability on these measures was associated with worse performance on traditional neuropsychological measures.

## Discussion

This study examined predictors of within-person variability on several repeated smartphone-based EMCTs in two US-based samples: a general adult sample and an older adult sample. In the general adult sample, participants who performed better *on average* exhibited greater consistency in their performance across most EMCT measures. Many tests demonstrated a quadratic association in which at lower levels of average performance IIV was greater overall, whereas at higher levels of average performance there was a more linear negative association. The opposite pattern of correlations was observed in working memory tests, possibly due to the difficulty of these tasks at higher levels of performance (i.e., no ceiling effects). These patterns largely replicated in the older adult sample with a broader range of cognitive performance, further supporting the robustness of these observed associations. Again, in this sample we observed that better average performance consistently associated with less variability with a similar quadratic association observed with many tests as in the adult sample. Additionally, some tests (e.g., Memory Matrix 18-Tile) showed lower variability in the lowest and highest performers with those in the middle demonstrating the greatest variability. Taken together, these findings suggest both linear and curvilinear associations between IIV and average performance. Individuals with lower levels of performance tended to either perform consistently poorly (e.g., as demonstrated by the Memory Matrix 18-tile) or show greater variability across time, likely depending on the difficulty of the task. However, generally, those with those with higher levels of cognitive performance did consistently well across most tests. Overall, our findings in two different samples indicate that associations with IIV vary by ability and task difficulty, which aligns with other research [36, 37]. However, this pattern has not been well explored due to the reliance on traditional cognitive measures that often exhibit ceiling effects.

These findings may suggest that the reliability of neuropsychological measures across time may vary as a function of average cognitive performance. In other words, this could imply that single test administrations may be less reliable at lower performance levels and assuming equal reliability across performance levels is potentially problematic. Many neuropsycholgoical tests, such as the Wechsler Adult Intelligence Scale-IV [38], the most widely used assessment for adults [19], do not examine differences in reliability across time at different levels of performance. Thus, further investigation into reliability across time at different performance levels may be a critical next step for improving the psychometric properties of single-administration neuropsychological assessments.

As noted in the introduction, increased variability is commonly observed across a range of populations with or at risk for cognitive impairment, thus we believe this finding reflects meaningful variability that has been established with other methods. This interpretation is further supported by the consistency of findings across both independent samples, including the older adult group, which was well-characterized using traditional neuropsychological assessments and demonstrated multiple associations between EMCT-derived IIV and conventional measures. Thus, we interpret our results as evidence that temporal variability captured by EMCT, particularly with further refinement, may offer clinically useful insights and ultimately supports the continued development of these tools for aging and cognitively at-risk populations.

Regarding the older adult sample, the finding that worse mean performance correlated with greater variability aligns with existing literature, which indicates that greater IIV is associated with an increased risk of developing age-related neurodegenerative disorders like AD [11, 39]. A systematic review of 22 studies found that greater IIV was a risk factor of cognitive impairment and mortality and that variability was predictive over and above traditional measures of central tendency [40]. Further, IIV is a marker of neurodegenerative diseases such as Parkinson’s disease [10] and Alzheimer’s disease and related dementias [41]. The current study contributes to the limited literature examining IIV across time in older adults using EMCT. For example Cerino et al. [25], found that EMCT-derived IIV in processing speed and short-term memory tasks was greater in persons with MCI, even when accounting for mean performance. Similarly, Aschenbrenner et al. 2024 found that IIV in older adults was not consistently associated with demographic factors like age or education [26]. While the literature on the relationship between age and IIV is mixed [42–44], our results suggest that increased age may not be associated with increased IIV, but rather that IIV may be more related to cognitive decline and underlying pathology. This possibility aligns with evidence that greater IIV reflects integrity of frontal gray matter density and function, as well as white matter volume [18]. Functional connectivity of the default mode and limbic networks are associated with IIV [45], indicating both structural and functional underpinnings of cognitive fluctuations.

Additionally, we found that greater IIV on EMCTs was associated with worse performance on several in-person neuropsychological assessments. This finding may further demonstrates that lower cognitive performance (as demonstrated by traditional neuropsychological assessments) is associated with greater within-person variability, independent of contextual and environmental factors, as this finding was observed in both standardized and unstandardized environments. Notably, variability on the shortest and easiest memory list measure displayed some of the most robust associations with traditional neuropsychological performance, suggesting that variability on easier tests may be sensitive to overall cognitive functioning. However, further refinement is needed to identify which measures hold the greatest clinical significance across different clinical populations.

Overall, we believe our findings suggest that EMCT-derived IIV could serve as a marker of cognitive vulnerability. Future work should explore its potential to identify people at very early or preclinical stages of disease. A few studies have examined the relationship between cognitive dispersion across traditional neuropsychological measures and AD pathology or MRI markers with mixed results [46–49]. For instance, one EMCT study showed that genetic AD risk (i.e., presence of an *APOE* e4 allele) was associated with greater variability on EMCT [26], and a follow-up study by the same group found that within-trial variability was associated with genetic AD risk. Another promising direction is that EMCT derived IIV may also correlate and/or predict everyday functioning and quality of life, which neuropsychological test scores do not consistently predict [50]. Cognitive dispersion on traditional measures has been associated with future functional decline [51] and worse current everyday functioning [52]. Greater IIV may reflect less consistent cognitive abilities in real-world settings and thus correlates with worse real-world functioning (e.g., occupational, social).

This study has several limitations. First, the absence of traditional neuropsychological testing within the general adult sample restricts the scope of our findings. Therefore, we cannot rule out the presence of objective cognitive impairment in this group. Additionally, the generalizability of our results may be limited, particularly within the older adult group, which was predominantly composed of non-Hispanic white individuals with higher levels of education. Our analyses were also restricted to monolingual English speakers within the U.S. Future research should adopt more inclusive sampling strategies and investigate IIV across different languages and cultural backgrounds. Another limitation is the relatively low number of EMCT administrations (i.e., four per task) used to estimate IIV. While prior IIV work has used a higher number of administrations [24], we prioritized feasibility to align with the demands of larger-scale studies and potential clinical applications, where repeated assessments over extended periods may not be feasible. Although our findings suggest that, across two different samples, meaningful variability patterns can be detected even with a limited number of trials, future research should examine the minimum number of administrations needed to yield stable and generalizable IIV estimates across populations and cognitive domains. Additonally, while we covaried for smartphone type, this may not fully account for known software and hardward differences.

In conclusion, this research highlights the potential usefulness of EMCT for assessing within-person variability, offering a valuable tool for further investigating several populations prone to cognitive fluctuations and groups with subtle impairments. A promising area for future exploration is to explore whether EMCT-derived IIV can predict long-term outcomes. If successful, EMCT could be used clinically to monitor individuals who appear cognitively normal according to traditional neuropsychological testing but may still be at risk for decline, facilitating early detection and intervention.

## Supplementary information

Supplemental Table 1.

Supplemental Table 2.

## Data Availability

The adult sample data has been submitted to the ICPSR repository and is available for download and at https://www.icpsr.umich.edu/web/pages/ICPSR/index.html with appropriate IRB approval. The older adult sample data has been submitted to the National Data Archive under the collection title: Introspective Accuracy, Bias, and Everyday Functioning in Severe Mental Illness.
