# Supplementary material for: Exploring the relationship between mean performance and within-person variability on smartphone-based cognitive testing in adults across the lifespan
Source: NPP Digit Psychiatry Neurosci. 2025 Jun 19;3:15. doi: 10.1038/s44277-025-00036-x (PMC12176645; doi:10.1038/s44277-025-00036-x)
Supplement: Supplementary file 1 — Supplemental Table 1. [file 44277_2025_36_MOESM1_ESM.docx]

**Supplemental Table 1.** Additional test statistics examining mean performance and individual variability on eight ecological momentary cognitive tests in the adult sample

|  | Beta | 95% CI | t ratio | *p*-value |
| --- | --- | --- | --- | --- |
| Memory List (log adjusted) | | | | |
| Linear | -0.033 | -0.039, -0.027 | -10.75 | <0.0001 |
| Quadratic | -0.0010 | -0.0015, -0.0005 | -4.33 | <0.0001 |
| Quick Tap 1 (log adjusted) | | | | |
| Linear | -0.050 | -0.060, -0.040 | -10.14 | <0.0001 |
| Quadratic | -0.0008 | -0.0011, -0.0005 | -4.79 | <0.0001 |
| Quick Tap 2 | | | | |
| Linear | -0.102 | -0.142, 0.062 | -4.95 | <0.0001 |
| Hand Swype (log adjusted) | | | | |
| Linear | -0.042 | -0.050, -0.034 | -9.94 | <0.0001 |
| Quadratic | -0.0004 | -0.0009, -0.0001 | -2.03 | 0.0434 |
| Odd One Out (log adjusted) | | | | |
| Linear | -0.043 | -0.052, -0.034 | -9.35 | <0.0001 |
| Quadratic | -0.0011 | -0.0017, -0.0006 | -4.21 | <0.0001 |
| CopyKat | | | | |
| Linear | 0.195 | 0.154, 0.236 | 9.31 | <0.0001 |
| Memory Matrix | | | | |
| Linear | 0.060 | 0.015, 0.106 | 2.63 | 0.0090 |
| Matching Pair | | | | |
| Linear | 0.017 | -0.026, 0.059 | 0.77 | 0.3788 |
