# Supplementary material for: Exploring the relationship between mean performance and within-person variability on smartphone-based cognitive testing in adults across the lifespan
Source: NPP Digit Psychiatry Neurosci. 2025 Jun 19;3:15. doi: 10.1038/s44277-025-00036-x (PMC12176645; doi:10.1038/s44277-025-00036-x)
Supplement: Supplementary file 2 — Supplemental Table 2. [file 44277_2025_36_MOESM2_ESM.docx]

**Supplemental Table 2.** Additional test statistics examining mean performance and individual variability on eight ecological momentary cognitive tests in the older adult sample

|  | Beta | 95% CI | t ratio | *p*-value |
| --- | --- | --- | --- | --- |
| Memory List 6-Word (log adjusted) | | | | |
| Linear | -0.196 | -0.254, -0.146 | -7.91 | <0.0001 |
| Quadratic | -0.008 | -0.012, -0.004 | -4.34 | <0.0001 |
| Memory List 12-Word | | | | |
| Linear | -0.251 | -0.340, -0.152 | -5.06 | <0.0001 |
| Memory List 18-Word | | | | |
| Linear | -0.095 | -0.190, -0.001 | -2.01 | 0.0486 |
| Memory Matrix 12-Tile (log adjusted) | | | | |
| Linear | -0.413 | -0.515, -0.311 | -8.07 | <0.0001 |
| Quadratic | -0.044 | -0.060, -0.028 | -5.44 | <0.0001 |
| Memory Matrix 18-Tile | | | | |
| Linear | -0.035 | -0.086, 0.015 | -1.41 | 0.1628 |
| Quadratic | -0.014 | -0.022, -0.006 | -3.58 | 0.0006 |
| Color Trick Meaning to Meaning (log adjusted) | | | | |
| Linear | -0.724 | -0.851, -0.598 | -11.55 | <0.0001 |
| Quadratic | -0.152 | -0.192, -0.112 | -7.74 | <0.0001 |
| Color Trick Meaning to Color (log adjusted) | | | | |
| Linear | -0.394 | -0.507, -0.280 | -6.96 | <0.0001 |
| Quadratic | -0.034 | -0.050, -0.019 | -4.47 | <0.0001 |
| Color Trick Yes-No Mechanic (log adjusted) | | | | |
| Linear | -0.450 | -0.588, -0.312 | -6.53 | <0.0001 |
| Quadratic | -0.055 | -0.092, -0.017 | -2.92 | 0.0048 |
